# Systematic Evaluation of Biodegradation of Azo Dyes by Microorganisms: Efficient Species, Physicochemical Factors, and Enzymatic Systems

**DOI:** 10.3390/ijms26167973

**Published:** 2025-08-18

**Authors:** Domingo Cesar Carrascal-Hernández, Erney José Orozco-Beltrán, Daniel Insuasty, Edgar Márquez, Carlos David Grande-Tovar

**Affiliations:** 1 Departamento de Química y Biología, Facultad de Ciencias Básicas, Universidad del Norte, Barranquilla 080020, Colombia; insuastyd@uninorte.edu.co (D.I.); ebrazon@uninorte.edu.co (E.M.); 2 Grupo de Investigación de Fotoquímica y Fotobiología, Programa de Química, Universidad del Atlántico, Carrera 30 No. 8–49, Puerto Colombia 081007, Colombia; eorozco1003@gmail.com

**Keywords:** azo dyes, bioremediation, enzymatic degradation, wastewater treatment

## Abstract

Modern culture, strongly influenced by the growth of sectors such as the fashion and textile industries, has generated an environmental trend that is difficult to reverse. It is estimated that between 60 and 70% of the dyes used in these sectors are synthetic, which offer great versatility, a low cost, and a broad spectrum of colors, making them indispensable in many sectors. Among these synthetic dyes, azo dyes stand out due to their excellent chromophoric properties, structural stability, and ease of synthesis. However, these compounds are considered xenobiotics with a strong recalcitrant potential. This review article comprehensively examines the biodegradation potential of azo contaminants by microorganisms, including bacteria, fungi, microalgae, and consortia, using the PRISMA 2020 methodology. In this regard, this study identified 720 peer-reviewed articles on this topic that are outstanding. The analysis of these studies focused on the effect of parameters such as pH, temperature, and exposure time, as well as the enzymatic degradation pathways associated with the degradation efficiency of these contaminants. For example, the results identified that microorganisms such as *Meyerozyma guilliermondii*, *Trametes versicolor*, *Pichia kudriavzevi*, *Chlorella vulgaris*, and *Candida tropicalis* possess significant potential for degrading azo dyes (up to 90%). This degradative efficiency was attributed to the high enzymatic activity that cleaves the azo bonds of these contaminants through specialized enzymes, such as azoreductases, laccases, and peroxidases. Furthermore, the results highlight synergistic effects or metabolic cooperation between species that enhance the biodegradation of these contaminants, suggesting an eco-friendly alternative for environmental remediation.

## 1. Introduction

The expansion and strengthening of global economic matrices have improved the quality of life for people and have also fostered the development of industrial sectors, such as the textile and fashion industries, which have a profound influence on modern culture. These sectors exert environmental pressure that is difficult to control due to the use of synthetic dyes in dyeing processes [1,2,3]. It is well known that several industrial sectors with a strong influence on modern culture are predominant in the use of synthetic dyes such as azo dyes [4]. The projections suggest that the global market for these dyes could reach USD 14.8 billion by 2032, underscoring not only the sector’s growth, but also the escalating environmental burden associated with dye discharge [5]. A critical problem lies in the inefficiency of dye fixation processes. For example, several prominent studies have reported that the annual production of synthetic dyes worldwide is around 7 × 10^7^, of which the textile industry alone uses more than 10,000 tons of these dyes for dyeing processes that are often inefficient (the dyes do not fully fix to the fibers), resulting in the discharge of between 15 and 50% of the dyes that do not bind to fibers into wastewater [4,6,7,8,9].

Industrial discharges with high concentrations of azo dyes have a significant impact on aquatic ecosystems. When they enter water bodies, these compounds increase the total organic carbon (TOC) levels, an indicator that reflects the sum of biodegradable and non-biodegradable organic matters present in the environment. This increase is due to the high chemical stability and structural strength of the dyes, which can disrupt essential biochemical processes, such as microbial respiration, photosynthesis, and the nitrogen cycle [9,10]. From a water quality perspective, azo dyes affect biochemical oxygen demand (BOD) and chemical oxygen demand (COD) parameters in several ways. BOD, used to determine the amount of oxygen required by microorganisms to decompose the biodegradable fraction of organic matter, typically increases moderately in the presence of these dyes, given their limited biodegradability and structural stability [11,12]. On the other hand, COD, which quantifies the oxygen required to chemically oxidize all organic matter (including the recalcitrant fraction), increases considerably [13]. This disparity demonstrates that azo dyes contribute more to the chemical load than to the biological load of the system, making their treatment difficult using conventional purification methods [14].

Furthermore, changes in parameters such as pH also have a profound impact on water quality. Many azo dyes contain sulfonate groups (-SO_3_Na) in their structure. These acidify the water, disrupting the ecosystem balance, affecting the solubility of metals and other substances, and increasing the toxicity of other pollutants [15]. Chromophore groups in these dyes enhance light absorption, which prevents or limits the penetration of sunlight, hindering photosynthetic processes in species such as algae and aquatic plants [16,17].

The chromophoric behavior of azo dyes is intricately modulated by the presence of electron-donating and electron-withdrawing substituents flanking the azo bond. This electronic interplay enables the precise tuning of both color intensity and solubility, allowing chemists to tailor dyes for specific substrates and industrial applications [18]. Their structural complexity and resistance to biodegradation have positioned them as a growing concern in aquatic and terrestrial ecosystems, where they contribute to long-term contamination and pose challenges for conventional wastewater treatment technologies [19].

This resilience becomes particularly problematic under anaerobic conditions, such as those found in sediment layers or within the gastrointestinal tracts of aquatic organisms. In such environments, the azo bonds are susceptible to reductive cleavage, leading to the formation of aromatic amines, many of which are recognized for their carcinogenic, mutagenic, and endocrine-disrupting properties [20,21,22].

Among the various strategies employed for textile effluent remediation, physical methods are commonly used due to their operational simplicity and low cost in removing chromophoric compounds such as azo dyes [23]. The most common methods include filtration processes (using membrane systems), adsorption (e.g., using activated carbon), and oxidation methods [24]. More robust prototypes employing nanostructured materials (e.g., graphene oxide and magnetic biochar) have been reported; their efficiency is limited, and they involve high costs [25,26,27,28].

Micro/nanofiltration is an attractive and common method for treating effluents with high azo dye loading. This technique is based on the removal of contaminants through size exclusion and electrostatic attraction that retains the dyes [29]. Membrane fouling requires persistent maintenance, which poses a prohibitive barrier to efficient removal of these contaminants [30,31].

Good degradation results have been reported with azo dyes through photocatalytic oxidation processes with TiO_2_ and/or ZnO. This process releases reactive oxygen species (ROS) such as OH, which are efficient for the mineralization of azo dyes, but affect other systems and species in the environment, generating another problem that is difficult to solve [32,33]. In response to this challenge, a range of biotechnological strategies has been explored. Notably, microbial strains such as *Streptomyces lavendulae*, *Streptomyces cyaneus*, and *Marinomonas mediterranea* have demonstrated promising capabilities in degrading azo dyes [34].

These biotechnological strategies are not only environmentally benign, but also economically viable, making them particularly attractive for large-scale applications in resource-constrained settings [35]. Biosorption enables the passive binding of dyes to microbial biomass, while enzymatic degradation (mediated by oxidoreductases such as laccases, peroxidases, and azoreductases) targets the chromophoric structures directly, often leading to complete mineralization. Synergistic enhancement of these mechanisms has been reported, which enhances the mineralization of these contaminants, while preventing the production of more toxic metabolites, representing an improvement in the treatment of dye-contaminated effluents [36].

A biotechnological approach demonstrated in several studies is the biosorption of azo pollutants; several microorganisms with the potential to degrade azo dyes have been reported [37]. It is well known that cell wall composition plays a fundamental role in the assimilation of these dyes; that is, walls rich in lipid compounds and heteropolysaccharides improve degradation efficiency because these macromolecules present groups, such as amino (–NH_2_, –NH_3_^+^), carboxyl (–COO^−^), phosphate (PO_4_^2−^) and hydroxyl (–OH), act as active sites for molecular recognition and complex formation with contaminants [38,39,40]. In addition, microorganisms present enzymatic machinery (azoreductase enzymes, which use reducing cofactors, such as NADH, NADPH, and FADH, to initiate electron transfer reactions) of high efficiency in the degradation of these dyes. This dual functionality (biosorption together with enzymatic degradation) positions microbial systems as versatile agents in the bioremediation of dye-laden effluents [41,42].

The eukaryotic microorganisms not only demonstrate resilience in the face of physicochemical stressors, but also possess highly effective mechanisms for removing xenobiotic compounds, including azo dyes. Their capacity to couple biosorption with enzymatic degradation has proven particularly advantageous, as the synergistic interplay between these pathways often results in enhanced decolorization and detoxification efficiencies [37,43,44]. Environmental parameters, such as pH and temperature, are essential for degradation efficiency because they modulate the assimilation and degradation potential. That is, *Candida albicans*, at an acidic pH (around 2.5 and room temperature), efficiently degrades Direct Violet 51 [38]. Similarly, *Candida tropicalis* is more efficient at slightly lower pH levels (around 4 and 43 °C), which underlines the need to control these parameters to favor its degradation efficiency [45].

This systematic review, conducted in accordance with the PRISMA (Preferred Reporting Items for Systematic Reviews and Meta-Analyses) guidelines, offers a critical and integrative perspective on recent peer-reviewed research concerning microbial bioremediation of azo dye-contaminated wastewater. The review explores how key operational parameters (such as pH, temperature, dye concentration, and exposure time) influence the efficiency of microbial degradation. It also delves into the enzymatic pathways that facilitate these processes. Special attention is given to identifying the most effective microbial species reported to date, with a focus on understanding the biochemical mechanisms that underpin their degradative capabilities.

## 2. Methodology

**Figure 1:**
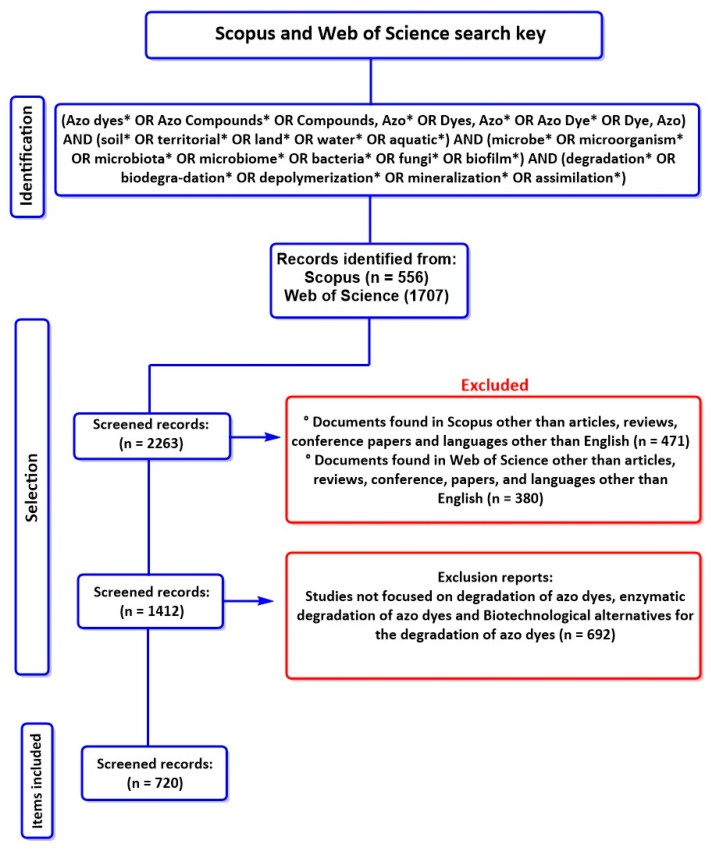
PRISMA methodology for searching and screening information. * characters are added to search terms to indicate plural terms.

This systematic review was developed in alignment with the PRISMA framework (see Figure 1) [46]. Our study identifies various operating conditions that favor or enhance the degradation efficiency of azo dyes. Similarly, the microbial strains with the highest efficiency in degrading these pollutants were comprehensively reviewed. This is important because it enables the design of innovative, highly efficient biotechnological strategies for treating effluents contaminated with these dyes.

To ensure methodological rigor and comprehensive coverage, the PICO strategy (Population/Problem, Intervention, Comparison, Outcome) was employed to structure the literature search. Table 1 outlines the development of the search key, which integrated Boolean operators, DeCS descriptors [47], and MeSH terms [48], to maximize the relevance and scope of retrieved studies [49]. The final search strategy was applied to Scopus and Web of Science (WoS), two databases recognized for their breadth and scientific reliability [50,51].

A total of 1707 scientific articles were initially identified in Web of Science and 556 in Scopus through an exhaustive bibliographic search using a Boolean combination of descriptors related to azo dyes, environmental matrices (soil and water), and microbial degradation pathways. The selection criteria consisted of selecting recent articles because it is relevant to report and discuss updated physical and biotechnological methods and/or alternatives for the degradation of azo dyes; the most recent articles on these technologies will include novel advances and improvements in the limitations presented in previous studies. Likewise, the search was limited to the English language because this is the universal language in which research is published. All keywords unrelated to the objective of this research were eliminated, and duplicate reports were eliminated to avoid information bias. The selection of the most notable results based on their approach, methodological relevance, and potential for industrial scalability is summarized in Figure 1.

The investigation of highly efficient biotechnological alternatives for the degradation of azo dyes is a dynamic field that has received growing interest in recent years, with microorganisms playing a central role in these studies. These microorganisms belong to diverse microbial communities, such as adapted microbiomes and biofilms, that are highly resistant to extreme environments. Notably, several studies have reported the complete mineralization of azo dyes under optimized conditions, highlighting the potential of microbial consortia to assimilate dye-derived intermediates [52,53,54].

## 3. Results and Discussion

### 3.1. Azo Dyes

There are two predominant types of dye, synthetic and natural, of which azo dyes are of particular interest to industries operating worldwide. These dyes are attractive because they exhibit excellent chemical stability, ease of synthesis, low costs, and have groups in their structures that allow for a wide range of structural versatility [16]. A distinctive feature of these compounds lies in the azo bond (–N=N–), which fulfills an important function. It acts as chromophores and joins various aromatic and/or heterocyclic compounds, generating conjugated systems that give rise to a wide range of shades; in addition, these conjugated systems present a strong absorption in the visible spectrum giving rise to intense colors [55]. These particularities make azo dyes indispensable for the textile and fashion industries [56].

**Figure 2:**
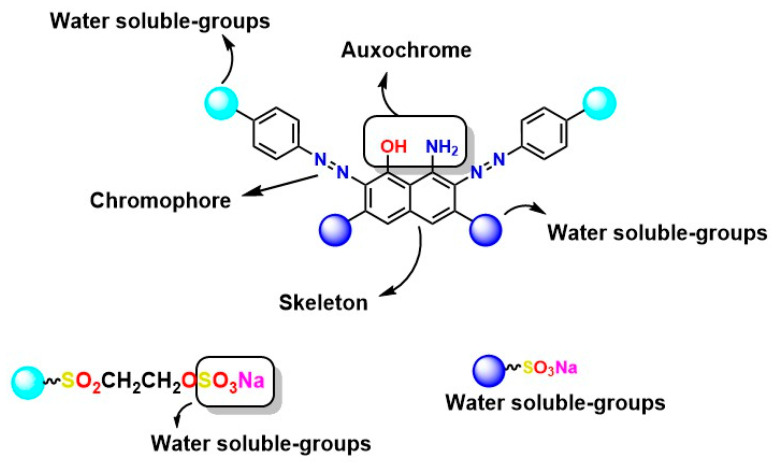
Chemical structure of azo dyes: two-dimensional representation of azo dye with groups in its structure that give it attractive properties.

The synthesis of azo compounds occurs through coupling reactions between various aromatic diazonium salts and activated nucleophiles, resulting in multiple structures depending on the number of units (N=N): monoazo, diazo, or polyazo [57]. Some common coupling agents include naphthol derivatives and sulfonated aromatic compounds such as J acid (2-amino-5-naphthol-7-sulfonic acid), H acid (1-amino-8-hydroxynaphthalene-3,6-disulfonic acid), and gamma acid [58]. The reaction proceeds via an electrophilic aromatic substitution mechanism, offering considerable structural flexibility through the strategic selection of precursors and reaction conditions, as illustrated in Figure 2.

Azo dyes are classified according to several criteria, including water solubility, dyeing behavior, number of azo linkages, and the nature of the substrate to which they are applied [35]. This versatility allows for them to be used across a wide range of materials, from natural fibers like cotton, silk, and wool to synthetic polymers, such as polyester, acrylics, and polyamides. Beyond textiles, azo dyes are also incorporated into inks, varnishes, lacquers, food additives, pharmaceuticals, and cosmetic formulations, reflecting their broad industrial relevance [35,59].

Azo dyes are commercially available in various physical forms (powders, liquids, and pastes) formulated according to their intended application [60]. Their solubility and dyeing behavior vary widely; acidic azo dyes, often containing sulfonate groups, are water-soluble, and suited for protein and polyamide fibers, while basic (cationic) dyes are typically applied to modified acrylics and synthetic textiles [56]. Direct dyes used primarily on cellulosic fibers require no mordants and are applied from aqueous solutions with electrolytes. In parallel, dyes with minimal solubility (such as disperse dyes) are designed for specific applications; that is, they are used in hydrophobic polyester and nylon-based fibers, which require particular pressure and temperature conditions (usually high temperatures) for greater fixation [58]. For this reason, the frequent use of these dyes is of concern in poorly treated effluents or those without rigorous pretreatment, given that they have been designed to be stable under extreme conditions.

**Figure 3:**
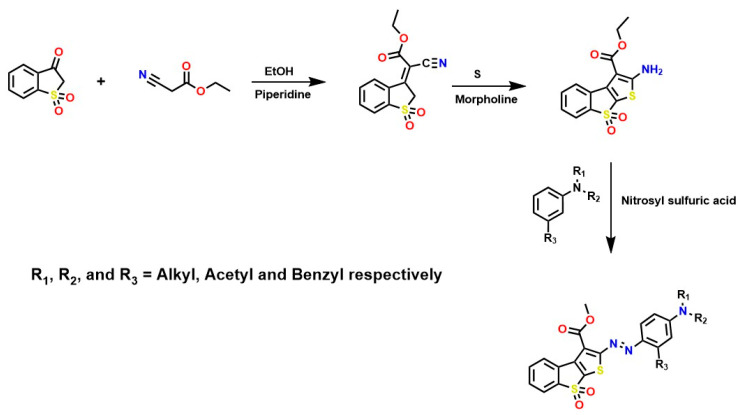
General scheme for synthesis of azo compounds.

Furthermore, innovative synthesis methods for obtaining functionalized dyes have been reported [61]. For example, the Gewald reaction is widely applied for dye synthesis [62,63]. That is, the condensation reaction between benzo-thiophene-3(2H)-one-1,1-dioxide and ethyl cyanoacetate, followed by diazotization using nitrosylsulfuric acid and N,N-dialkyl-substituted aryl amines (for acid coupling), has been reported to give rise to dyes of high interest in industrial sectors, as shown in Figure 3 [61,64]. However, these compounds can cause chronic toxicity, primarily through ingestion, which may occur when consuming foods highly contaminated with these compounds [65].

#### 3.1.1. Azo Dyes and Their Structural Versatility

**Figure 4:**
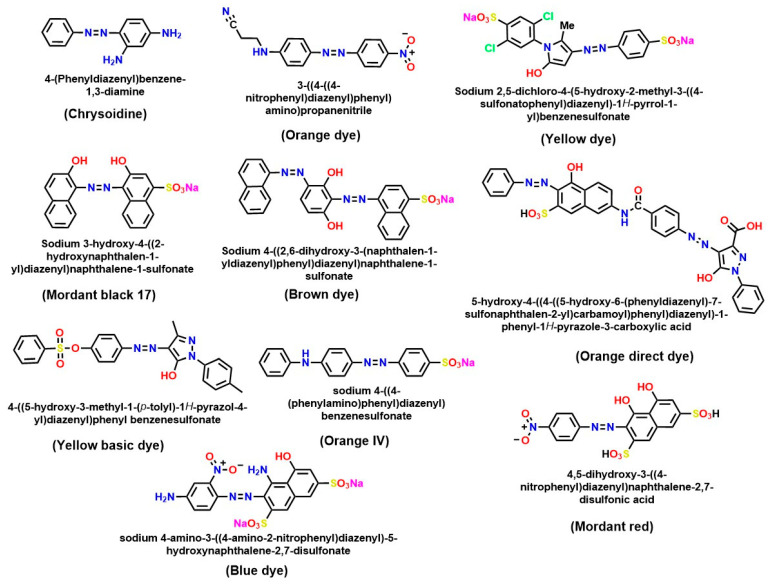
Chemical structures of azo dyes, which are common in industries that use dyes.

The environmental persistence of these compounds is closely tied to their chemical structure. The presence of functional groups influences properties such as polarity, solubility, thermal stability, and lightfastness (see Figure 4). Electron-donating substituents (e.g., –NH_2_ and –OH) tend to enhance biodegradability under reducing conditions, whereas electron-withdrawing groups (e.g., –NO_2_) confer greater stability and resistance to degradation [66,67]. Additionally, some azo dyes form coordination complexes with transition metals, such as chromium, copper, and cobalt, which improves dye fixation, but simultaneously increases toxicity and environmental recalcitrance [68].

Once released into the environment, azo dyes exert a series of harmful effects. That is, the degradation of these compounds generates metabolites that are even more toxic than the original dyes. These metabolites present higher toxicity levels (LD_50_) than the original molecules, which are carcinogenic, mutagenic, or endocrine disruptors, with the potential to bioaccumulate in organisms and biomagnify through food chains [69,70].

The chemical structures of several chemical dyes widely used in various industries are represented in Figure 4. These dyes are attractive due to their stability in extreme situations and chemical versatility. Due to the toxicity of azo dye metabolites, it is essential to use innovative and efficient strategies for their transformation and/or bioremediation to ensure more sustainable wastewater treatment [71,72].

#### 3.1.2. Toxicological Potential of Azo Dyes

Despite their industrial relevance, azo dyes remain poorly regulated in many regions, particularly where environmental legislation is weak or underdeveloped [3,73]. This regulatory gap has led to the widespread and often indiscriminate use of these dyes, resulting in the direct discharge of structurally complex dyes into natural ecosystems. Many of these compounds contain sulfonate, nitro, halogen, or heterocyclic groups that enhance their chemical stability and resistance to degradation, making their removal through conventional treatment methods more complicated [74].

More concerning is that environmental degradation (especially under anaerobic conditions) often leads to the formation of toxic byproducts [75]. Microbial azoreductases cleave the azo bond (–N=N–), releasing aromatic amines, such as β-naphthylamine, aniline, and *p*-phenylenediamine, several of which are classified by the International Agency for Research on Cancer (IARC) as confirmed carcinogens [76,77]. Dyes such as Reactive Red 195, Acid Orange 7, and Reactive Black 5, though widely used for their dyeing efficiency, are now recognized as precursors to these hazardous metabolites [78,79,80,81].

**Figure 5:**
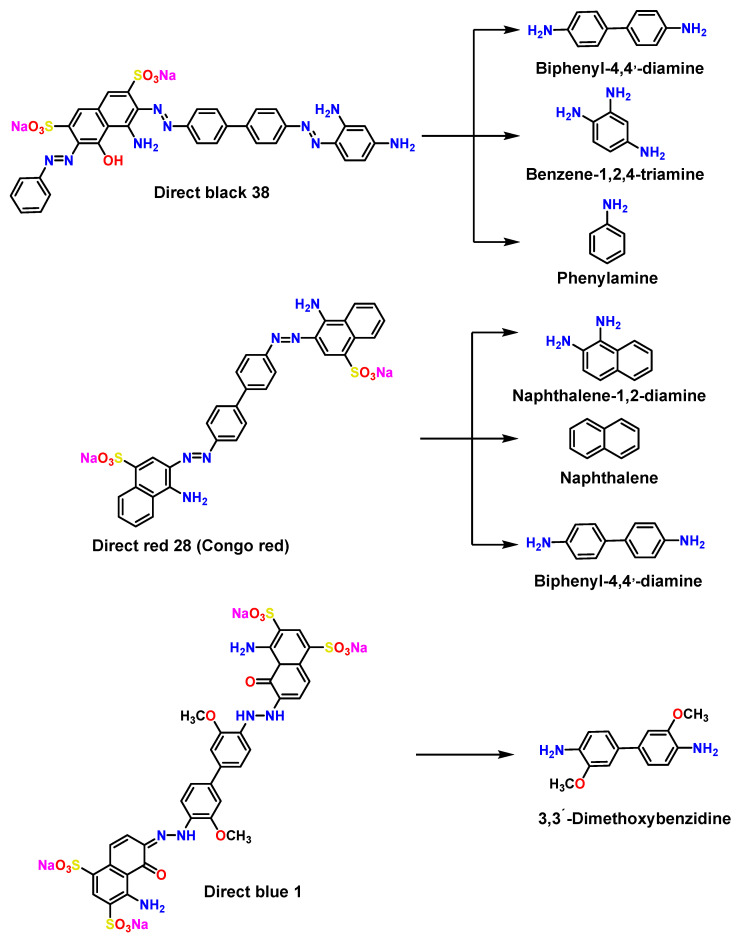
Chemical structures of common dyes and their decomposition products that hurt the environment.

Ecotoxicological assessments often overlook these transformation products, instead focusing on the parent compounds under controlled conditions that fail to reflect environmental complexity accurately. Some studies have shown that photolytic, chemical, and microbial degradation can yield more toxic and bioactive derivatives [82]. The resilience of azo contaminants depends largely on extrinsic dynamic factors, such as oxygen availability, light exposure, temperature, pH, and the abundance of compounds involved in redox reactions. In addition, the molecular characteristics involve the number and position of bonds –N=N– and the chemical nature of substituent groups [83]. Microbial consortia have been reported to be effective in degrading azo dyes because they possess enzymes capable of metabolizing these bonds (as shown in Figure 5). However, most are severely affected by these contaminants, resulting in the alteration of the ecological balance [84,85].

Several studies have shown that some dyes classified as “non-toxic” in acute tests (such as Acid Orange 7) generate carcinogenic metabolites under natural conditions [72]. This situation generates a false sense of security. Coupled with inadequate infrastructure for the treatment of wastewater contaminated with these dyes, they continue to aggravate environmental and public health risks [86].

Intrinsic factors, on the other hand, are determined by the molecular structure of the dye. The number and position of azo bonds, the nature of the functional groups (donors such as –OH and –NH_2_ or attractors such as –NO_2_, –Cl, and –SO_3_H), and the presence of heteroatoms or condensed structures directly influence chemical reactivity and susceptibility to enzymatic biodegradation [87]. The microbial communities that encounter these dyes vary significantly in their response capacity. While some bacterial and fungal consortia have azoreductases, laccases, peroxidases, or monooxygenases capable of metabolizing azo compounds, other microbial populations can be suppressed or damaged, altering the ecological balance of the contaminated environment [69].

Given this situation, the lack of strict regulatory frameworks and toxicological assessment methodologies currently in place to ensure the proper management of highly contaminated industrial effluents does not guarantee effective management [88,89]. This demonstrates that the lack of methodological rigidity in evaluating the toxicity of synthetic dyes is generating significant environmental pressure, as they are freely released without appropriate prior treatment, despite being considered non-toxic [90].

As illustrated in Figure 5, certain widely used dyes (such as Direct Black 38, Direct Red 28, and Direct Blue 1) have been shown to be moderately toxic at 7600 mg/kg. In comparison, Direct Blue 1 is toxic at 6700 mg/kg, which is classified as class VI. The degradation of these contaminants does not necessarily neutralize their toxicity [37]. On the contrary, it leads to the formation of byproducts whose hazards persist, albeit to varying degrees [91]. A detailed analysis using the Protox 3.0 server [91] (a specialized tool for predicting chemical toxicity) allowed for the estimation of median lethal doses (LD_50_) of some of these compounds, expressed in mg/kg [92].

In the case of Direct Black 38, metabolites such as biphenyl-4,4′-diamine, benzene-1,2,4-triamine, and aniline were identified, with LD_50_ values of 205, 80, and 250 mg/kg, respectively. These data place them in the class III toxicity category, i.e., potentially toxic compounds if ingested in quantities between 50 and 300 mg/kg. Meanwhile, the byproducts of Direct Red 28 (naphthalene-1,2-diamine, naphthalene, and biphenyl-4,4′-diamine) exhibit more moderate toxicity, with LD_50_ values of 727, 316, and 205 mg/kg, respectively, classifying them in category IV (300 < LD_50_ ≤ 2000). Finally, Direct Blue 1 generates a byproduct (3,3-dimethoxybenzidine) with an LD_50_ value of 1920 mg/kg, also within class IV, suggesting relatively minor toxicity [92].

Given the enormous structural diversity of azo dyes (many of them proprietary formulations and confidential compositions), exhaustive toxicological studies for each compound are impractical [93]. In response to this limitation, the scientific community has begun to develop predictive models based on structure–activity relationships (SARs), computational toxicology, and environmental simulations, which allow for the prediction of toxic or persistent behaviors based on specific molecular characteristics [94,95,96].

The real danger posed by azo dyes is not limited to their visible presence as color pollutants [97]. Their greatest threat lies in the byproducts of the transformations these pollutants undergo in ecosystems; their conversion generates reactive, persistent, and often carcinogenic species, which increases their potential for contamination [98]. Addressing this challenge requires going beyond static toxicity models, adopting dynamic, integrative, and systemic approaches that capture the true complexity of the interactions between these synthetic molecules and the living systems that absorb, degrade, or suffer the consequences [99,100].

## 4. Conventional Methods for Treating Azo Contaminants: Principles, Challenges, and Perspectives for Environmental Remediation

In response to the environmental impacts caused by the release of synthetic dyes, particularly azo compounds, various remediation strategies based on physicochemical and biological processes have been developed [101]. These technologies aim to mitigate the toxicity, mobility, and persistence of these pollutants; however, each approach presents technical, economic, and environmental limitations that must be considered during their implementation [102].

**Figure 6:**
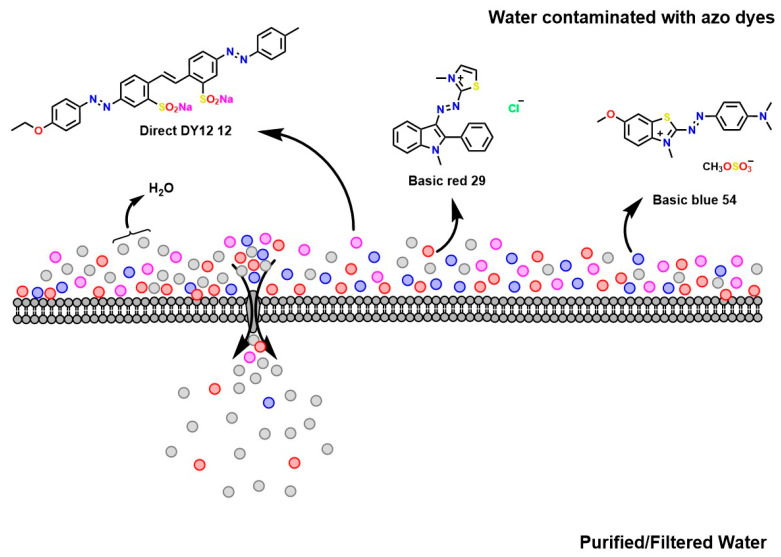
Mechanism of dye separation using membranes.

Among the most widely used physicochemical methods are membrane separation processes, such as reverse osmosis, ultrafiltration, and nanofiltration (see Figure 6) [37]. These systems rely on pressure and/or concentration gradients to force the passage of water or solvent through semipermeable membranes, retaining larger molecules, including dissolved dyes. Their effectiveness depends on the quality of the influent; the presence of organic matter, suspended solids, or fats can quickly saturate the membranes, reducing their efficiency [103]. For this reason, pretreatments such as coagulation and adsorption are commonly implemented to prevent scaling and extend the system’s useful life. Despite their high removal capacity, these technologies require considerable energy consumption and can generate concentrated waste streams, which pose a new management challenge [104].

**Figure 7:**
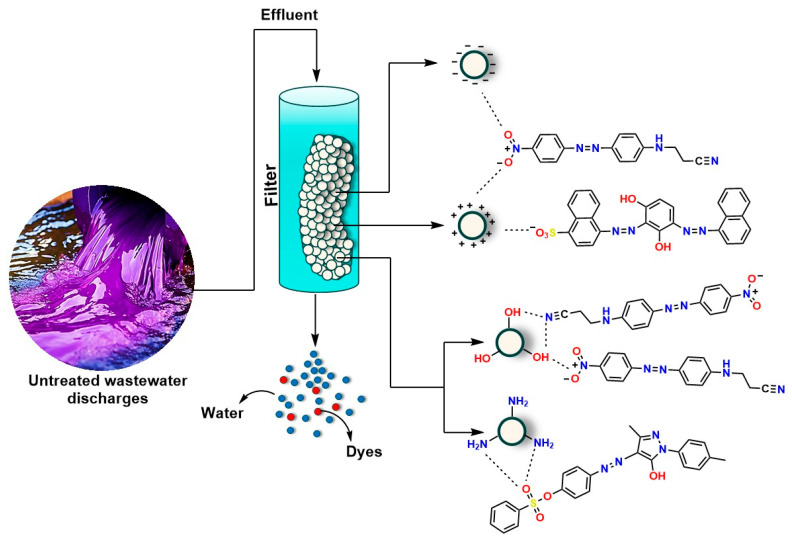
Ion exchange-mediated dye removal.

Ion exchange represents another alternative based on the affinity between charged functional groups of polymeric resins and the ionic groups of dyes (see Figure 7) [105]. This technique has proven effective for the selective removal of certain anionic or cationic dyes; its applicability is limited, as many industrial dyes have neutral or mixed structures that do not interact efficiently with the available resins. Furthermore, the process requires periodic regeneration of the exchange medium, which entails the use of saline solutions that can generate problematic liquid waste [106].

Coagulation–flocculation is a widely used technique in textile wastewater treatment for removing colloidal and insoluble particles. It involves the addition of chemical coagulants (such as aluminum or iron salts) that neutralize the surface charges of contaminants, allowing for their agglomeration and subsequent sedimentation. Although effective in removing turbidity and certain hydrophobic dyes, this technique generates chemical sludge that requires further treatment, increasing the disposal costs and increasing the risk of secondary pollution if not properly managed [105].

**Figure 8:**
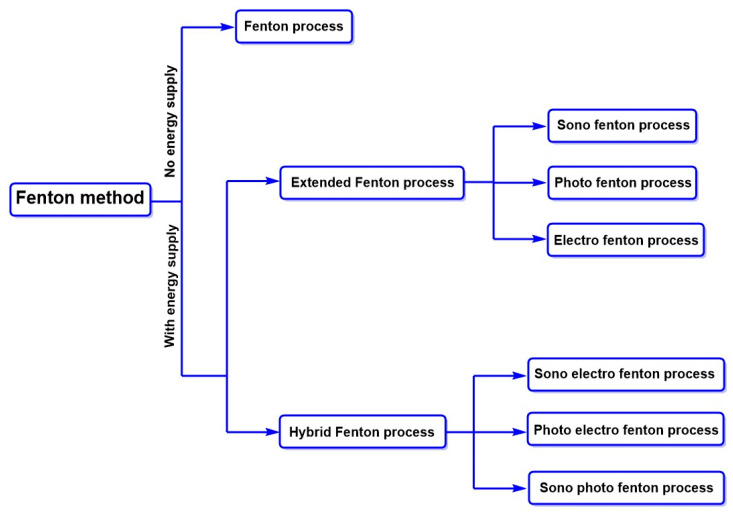
Classification of Fenton methods for degradation of dyes.

Some other methods involve advanced oxidation processes to produce hydroxyl radicals (•OH) as oxidizing agents. These radicals are known for their strong oxidative character, which makes them highly reactive chemical species in the presence of organic compounds [107]. They subtract hydrogen atoms or add hydrogen atoms to double bonds, giving rise to new oxidized species or intermediates, or even almost entirely mineralizing the organic compounds [108]. The more advanced methods for degrading azo dyes involve the use of potent oxidizing agents that produce reactive oxygen species [109]. For example, the Fenton method is beneficial for treating azo contaminants through their oxidative degradation using the redox couple Fe^2+^/Fe^3+^. This oxidative degradation generates less recalcitrant metabolites, representing an attractive approach for more efficiently degrading azo dyes. Figure 8 summarizes the standard Fenton process methods reported for the degradation of azo dyes [109,110].

The Fenton method variants has four essential steps: (I) acidification of the reaction medium to an optimal pH of approximately 3.0; (II) oxidative degradation of the target pollutants; (III) neutralization of the treated effluent; and (IV) a final coagulation–flocculation phase aimed at removing residual solids and metal complexes [111]. The initial acidification step is particularly critical, as it establishes the physicochemical conditions necessary for the efficient generation and stabilization of •OH, the primary oxidative species in the process. Within this mildly acidic environment, both hydrogen peroxide (H_2_O_2_) and ferrous ions (Fe^2+^) exhibit enhanced reactivity and stability, thereby maximizing the oxidative potential of the system and ensuring the effective breakdown of recalcitrant contaminants [112].

Furthermore, the redox pair Fe^2+^/Fe^3+^ plays a crucial role not only in redox processes, but also as a coagulating agent. This physicochemical characteristic enhances its efficiency in the removal and degradation of azo structures [113]. This dual behavior highlights the versatility of the Fenton method for the treatment of highly contaminated industrial effluents. Its mechanism is described in Equations (1)–(4), where -R indicates the organic structure of any azo dye; these equations specifically show the reactive species directly involved in the degradation of these pollutants [114,115,116].

(1) $${Fe}^{2+}+H_{2}O_{2}\to {Fe}^{3+}\;{OH}^{-}+{OH}^{•}$$

(2) $$RH+{OH}^{•}\to R^{•}+H_{2}O$$

(3) $$R^{•}+{Fe}^{3+}\to R^{+}+{Fe}^{2+}$$

(4) $${Fe}^{2+}+{OH}^{•}\to {Fe}^{3+}+{OH}^{-}$$

Finally, heterogeneous photocatalysis utilizes semiconductors such as titanium dioxide (TiO_2_) that are activated by ultraviolet or visible radiation [117]. By absorbing light energy, these materials generate electron–hole pairs that react with water or oxygen present, producing reactive oxygen species that can degrade a wide range of organic pollutants. Although this technique is promising for its ability to mineralize dyes to CO_2_ and H_2_O completely, its efficiency depends on variables such as light intensity, the surface area of the catalyst, and the presence of interfering substances [118]. These technologies represent significant advances in mitigating azo dye pollution, but also highlight the need to integrate combined treatments and more sustainable approaches (Table 2). The development of hybrid systems, which incorporate physical, chemical, and biological processes, is emerging as a promising strategy for maximizing efficiency and minimizing collateral impacts in the remediation of complex effluents [119].

### Application of Biological Treatments in the Remediation of Industrial Effluents

A range of microorganisms have evolved metabolic strategies to degrade azo dyes, with genera such as *Pseudomonas*, *Bacillus*, *Enterococcus*, and *Saccharomyces* standing out for their enzymatic versatility [129]. The microbial enzymatic machinery is efficient in cleaving monoazo, diazo, and polyazo bonds from complex and persistent dye structures. For example, azoreductases are considered flavo-dependent enzymes. That is, they employ FMN and/or FAD as cofactors (FMN, flavin mononucleotide, which participates as an electron transporter in redox enzymes; FAD, flavin adenine, which participates in more complex redox reactions and can act as a redox mediator). These enzymes reduce the –N=N– bond through a highly efficient biocatalytic mechanism, transforming dyes into cyclic amines that can then be degraded in other metabolic pathways [130]. A notable example is AzRA (FMN-dependent), identified in *Bacillus* sp. B29 (PDB code: 3W77), which efficiently degrades Acid Red 88 and Orange I [131]. However, the interaction between dyes and microbial communities is far from neutral. Microbiological studies have shown that the presence of these compounds can significantly alter microbial assemblages. Beneficial species, such as nitrogen-fixing *Niveispirillum* spp., may be displaced by more tolerant opportunistic strains, disrupting key ecological functions, including nutrient cycling, organic matter decomposition, and overall ecosystem stability [132].

Given the growing environmental impact generated by effluents from the textile industry (particularly due to the presence of recalcitrant azo dyes), various bioremediation strategies have gained prominence as sustainable and effective alternatives for mitigation [133]. Among these, biomass-based treatments stand out, operating through biosorption and/or adsorption processes. However, microbial systems (comprising bacteria, filamentous fungi, yeasts, actinobacteria, and microalgae) offer the most significant potential for transforming and mineralizing these pollutants into harmless compounds, such as CO_2_ and H_2_O, and, in some cases, additional biomass, provided optimal environmental conditions are maintained [134].

Table 3 summarizes a representative selection of microorganisms capable of degrading azo structures, whose physicochemical properties (such as the high stability of the azo bond (–N=N–) and the presence of aromatic functional groups) hinder their spontaneous degradation in the environment [135]. This structural resistance makes azo dyes persistent pollutants with a high ecotoxicological risk. However, the phylogenetic diversity of the reported microorganisms allows us to infer the existence of multiple metabolic pathways capable of adapting to different environments (aerobic, anaerobic, and microaerophilic), which reinforces their applicability in wastewater treatment processes [136].

At the enzymatic level, these microorganisms exhibit distinct, but complementary mechanisms of action; bacteria play a central role under reducing conditions, where they catalyze azo bond cleavage using NADH/NADPH-dependent azoreductases as cofactors [137,138]. They can also express oxidoreductases, monooxygenases, and peroxidases, which facilitate the subsequent degradation of intermediate metabolites; filamentous fungi, on the other hand, use broad-spectrum ligninolytic enzymes, such as laccase, lignin peroxidase (LiP), and manganese peroxidase (MnP), capable of attacking complex aromatic compounds through non-specific oxidative mechanisms [139]. Yeasts have demonstrated versatility in aerobic systems, expressing azoreductases, laccase, LiP, and MnP, making them useful biotechnological tools in variable environments [140]. In the case of microalgae and cyanobacteria, their degradation capacity is linked to both surface adsorption processes and the production of specific enzymes (e.g., azoreductases), in addition to their interaction with light and CO_2_, which gives them additional advantages in photoautotrophic systems. Actinobacteria, particularly active in anoxic conditions, employ metabolic pathways based on anaerobic respiration and fermentation, utilizing alternative electron acceptors to oxygen to break azo bonds, thereby demonstrating effectiveness in environments where other species fail [141].

The versatility of both these biological and physicochemical mechanisms, as well as their synergistic effects, demonstrates the existing ecological and operational capacity for treating effluents contaminated with azo dyes. However, some limitations must be overcome to achieve greater efficiency. These limitations could be overcome synergistically with other species that overcome the limitations of other species in the presence of specific conditions or specific compounds. To achieve this, further research is needed on the synergistic effects of the enzymatic potential of appropriate taxa [142].

**Table 3:** A sample of microorganisms used for the degradation of azo dyes.

Type of Microorganism	Species	Treated Azo Dyes	Mechanism Involved	Conditions (pH, Temperature, and Presence or Absence of O_2_)	Efficiency (%)	Ref.
Bacteria	*Pseudomonas putida*	Reactive Red 120	Azoreductase	pH 7.4	92.6	[143]
		Reactive Black 5		35 °C	92.6	
		Reactive Blue 13		Anaerobic conditions	88.0	
	*Bacillus subtilis* (DY1KVG)	Azo dye mixtures: Reactive red, Reactive brown, Reactive black	Azoreductase	pH 7–8.5	87.3	[144]
				50–70 °C		
				Aerobic conditions		
	*Escherichia coli*	Basic Orange 2	Azoreductase	pH 4	89.8	[145]
				40 °C		
				Aerobic conditions		
	*Pseudomonas geniculata* Ka38	Methyl orange	Azoreductase	pH 7	89.0	[146]
				30 °C		
				Mixed conditions (aerobic/anaerobic)		
Filamentous fungi	*Oudemansiella canarii* (EF72)	Congo Red	Laccase	pH 5.5	80.0	[88]
				30 °C		
				Presence of O_2_		
	*Trametes hirsuta* D7	Acid Blue 29 Reactive Blue 4	Laccase	pH 4–5	86–90	[88]
				25 °C		
				Aerobic conditions		
	*Nigrospora sp*	Synazol Red HF-6BN	Ligninolytic enzymes	pH 5–7	85.0	
				25–30 °C		
				Aerobic conditions		
	*Trametes versicolor*	Direct Pink B	Manganese Peroxidase	pH 5.2	72.4	[147]
				29.6		
				Aerobic conditions		
	*Aspergillus terreus*	Acid Blue 29 Disperse Red 1 Congo Red	Ligninolytic enzymes	pH 7	92.7	[88,148]
				30 °C	90.5	
				Aerobic conditions	96.7	
	*Aspergillus niger*			pH 7	93.4	
				30 °C	84.2	
				Aerobic conditions	96.5	
	*Aspergillus flavus*			pH 7	92.4	
				30 °C	80.5	
				Aerobic conditions	96.3	
	*Aspergillus fumigatus*	Direct Pink B	Manganese Peroxidase	pH 7	91.8	[147]
				35 °C	95.5	
				Aerobic conditions	97.0	
Actinobacteria	*Streptomyces albidoflavus 3MGH*	Reactive Orange 122	Reductive enzymes (such as azoreductase) and oxidative enzymes (such as laccase)	pH 6	94.4	[149]
		Direct Blue 15		36 °C	86.3	
		Black Direct 38		Aerobic conditions	68.2	
	*Arthrobacter bambusae* DP-A9	Methyl red Brilliant black	Azoreductase Peroxidase Laccase	pH 7 30 °C Aerobic conditions	100	[150]
					100	
					100	
	*Dermacoccus nishinomyaensis* DP-D10				100	
					74.0	
					97.6	
	*Leifsonia shinshuensis* DP-L11					
	*Streptomyces maritimus (A011)*	Amido Black10B	Extracellular enzymes: peroxidases (lignin peroxidase, manganese peroxidase), Laccases	pH 7–9	85.4	[151]
				30–40 °C		
				Anoxic conditions		
Yeasts	*Geotrichum candidum*	Congo red	Manganese peroxidase, Lignin peroxidase	pH 5–6	85.4	[152]
				25–30 °C		
				Microaerophilic/anoxic conditions		
	*Sterigmatomyces haophilus* SSA1575	Reactive Black 5	NADH-dichlorophenol indophenol (NADH--DCIP). Reductase Lignin peroxidase (LiP).	pH 5	100	[153]
				30 °C		
				Aerobic conditions		
	*Meyerozyma guilliermondii* A4	Acid Red B	Azoreductase. NADH-DCIP reductase. Lignin peroxidase. Manganese peroxidase. Laccase	pH 6 35 °C Aerobic conditions	>97	[154,155]
		Acid Orange II				
		Acid Scarlet GR				
		Acid Red 3R				
		Reactive Brilliant				
		Red K-2G				
		Reactive Violet KN-4R				
		Reactive Yellow 3R				
	*Meyerozyma caribbica*	Acid Orange 7	Manganese peroxidase	pH 5–7	93.8	[155]
				28 °C		
				Aerobic conditions		
Microalgae	*Lychaete pellucida*	Reactive Blue 4	Biosorption	pH 8.0	96–97	[156]
		Reactive Red 120		25 °C	95–97	
		Brilliant Reactive Yellow 3G		The study reports photoautotrophic culture conditions with continuous light exposure and O_2_ supplementation at room temperature	96–97	
		Reactive Green 12				
	*Chlorella vulgaris*	Reactive Black 5	Azoreductase	pH 5–8	80	[157]
		Direct Blue 71		40 °C	78	
		Scattered Red 1		The study reports mixotrophic culture conditions, with controlled light exposure to enhance dye degradation	84	

On the other hand, it is essential to take into account the final destination and disposal of the microbial biomass generated in these processes because the accidental or intentional release of adapted microorganisms (or genetically modified to increase their degradative efficiency) into the environment can hurt native microbial communities; in addition, the appearance of strong odors can generate respiratory diseases if there are nearby populations [158]. For example, several studies have reported diverse metabolites present in the residual biomass generated in these microbial treatments for the treatment of wastewater contaminated with azo dyes, which can vary in toxicity, chemical structure, and persistence in the environment, depending on the type of microorganism, environmental conditions, and types of dye. In this sense, among the reported metabolites, aromatic amines are common, such as aniline, 2-naphthylamine, p-nitroaniline, 4-aminoazobenzene, and bezidine. These substances are considered carcinogenic and mutagenic with a strong potential for bioaccumulation in aquatic organisms [159]. Substituted phenols (generated by the oxidation of aromatic rings), such as 4-hydroxybenzene and 2,4-dinitrophenol, can be toxic to a wide range of organisms and can interfere with metabolic processes [160]. In addition, organic acids, such as benzoic acid and carboxylic acid derivatives, are considered less toxic, but can significantly alter the pH and affect the solubility of other substances [17].

In this context, it is recommended to be rigorous in the implementation of safe biomass disposal strategies; anaerobic digestion and controlled composting are attractive strategies for biomass treatment [161,162]. Similarly, it is essential to consider the relevant physical and biological containment measures to avoid the release of these microorganisms and prevent their negative impact on microbial communities, as well as the monitoring of collateral environmental consequences, such as the generation of strong odors that can cause respiratory diseases.

## 5. Enzymatic Systems in the Biodegradation of Azo Dyes: Reductive and Oxidative Mechanisms in Bioremediation

Microorganisms have established themselves as key players in various environmental biotechnology applications, particularly in the bioremediation of organic pollutants. This is due to their remarkable physiological properties, including rapid growth, the ability to synthesize degradative enzymes, and their remarkable metabolic versatility in transforming complex compounds, such as synthetic dyes, especially azo dyes [17,163].

These characteristics make microorganisms ideal tools for environmental decontamination processes. Several recent studies have confirmed that the effectiveness of various microorganisms in degrading azo dyes is based on the action of specialized enzymes, such as azo reductases and laccase. These enzymes catalyze the cleavage of the azo bond (–N=N–), which is considered key to the structural stability and toxicity of these compounds [164,165].

**Figure 9:**
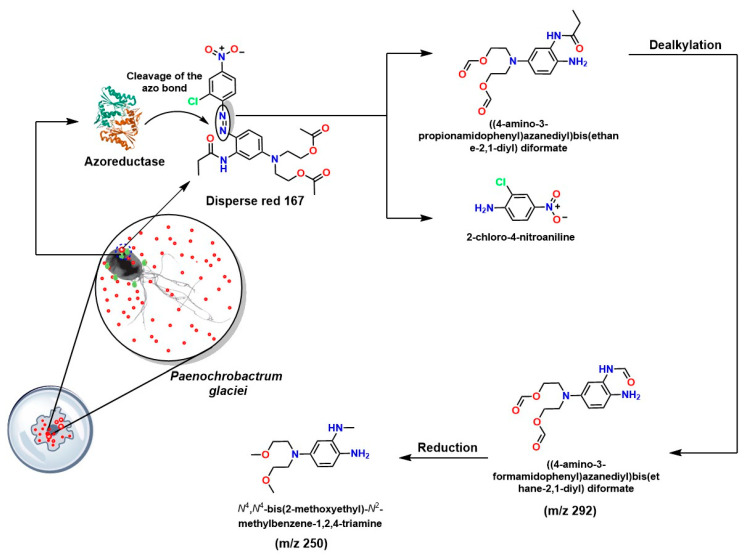
Mechanism of disperse red 167 degradation by Paenochrobactrum glaciei [166].

For example, the bacterial degradation of azo dyes involves complex enzymatic systems, in which both azoreductases and laccases play crucial roles. *Paenochrobactrum glaciei* has been documented to degrade the disperse red dye 167 through the coordinated production of laccase (oxidative) and azoreductase (reductive), as shown in Figure 9. Analysis by gas chromatography coupled with mass spectrometry (GC-MS) elucidated a metabolic pathway in which two intermediate products were identified after the cleavage of the azo bond: the first, 2-chloro-4-nitrophenylamine, and the second, an ethyl ester derived from a substituted aniline. These compounds emerge after the synergistic action of azoreductase and subsequent dealkylation reactions [166].

**Figure 10:**
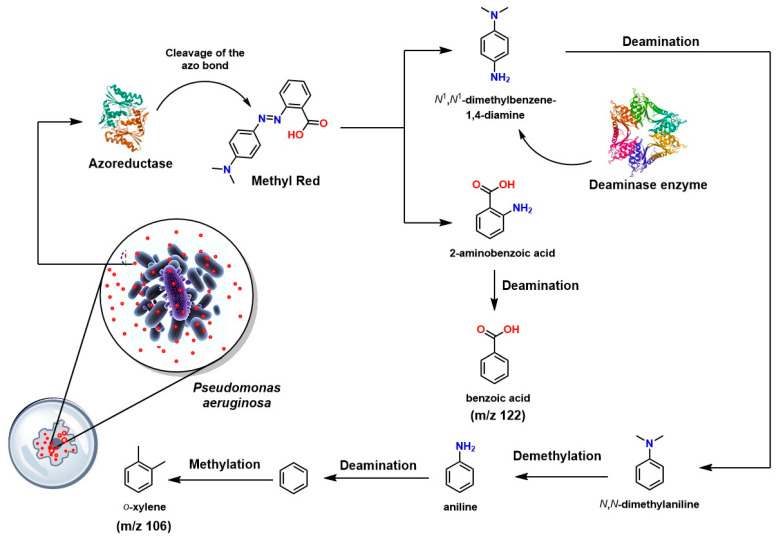
Mechanism of methyl red degradation by Pseudomonas aeruginosa [168].

In parallel, other studies have shown that *Pseudomonas aeruginosa* can degrade the dye methyl red through an enzymatic system centered on azoreductases, as shown in Figure 10. The reductive cleavage of the azo group produces two key metabolites: 2-aminobenzoic acid and N,N-dimethylaminobenzene [167]. The former undergoes enzymatic deamination to form benzoic acid, while the latter is initially transformed into N,N-dimethylbenzene, which can be demethylated to form aniline. Aniline, in turn, can be deaminated to generate benzene (Figure 9). Finally, through methylation reactions (possibly catalyzed by S-adenosylmethionine and tetrahydrofolate), benzene is converted into *o*-xylene. These transformations were confirmed by GC-MS, validating the ability of *P. aeruginosa* to efficiently destructurize and detoxify the dye [168].

**Figure 11:**
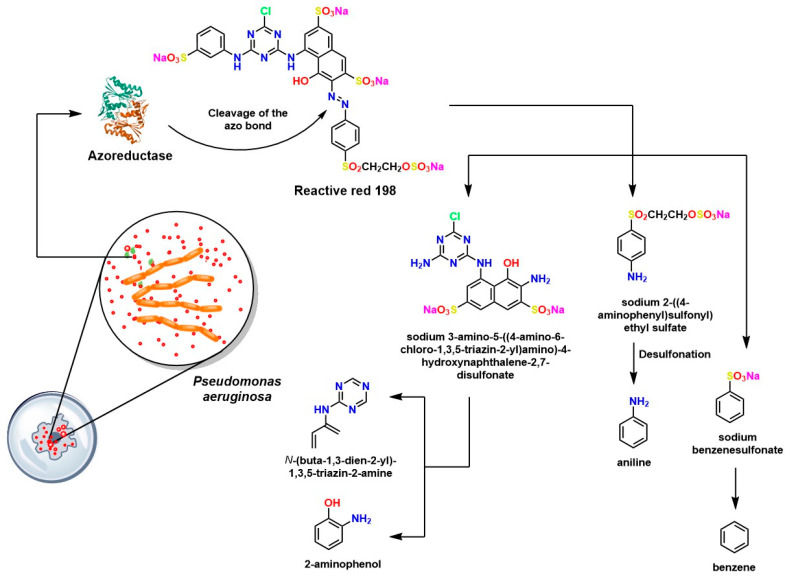
Proposed mechanistic pathway of reactive red (RR 198) degradation by *Bacillus cereus* SKB12 [170].

Several notable investigations have demonstrated the good efficiency of laccases and lignin peroxidases in degrading azo bonds, such as Reactive Red 198, also known as RR198. For example, bacterial strains such as *Bacillus cereus* SKB12 and *Enterobacter hormaechei* SKB16 have been reported to be capable of synthesizing laccases, lignin peroxidases, and azoreductases [169]. This characteristic confers on them the ability to biodegrade azo bonds, as shown in Figure 11. These microorganisms are attractive for the bioremediation of azo dye contamination due to their strong potential for cleaving N=N bonds. Furthermore, rigorous toxicity studies have shown that the metabolites of the dyes biodegraded by these species are non-toxic. That is, aromatic amines that are normally toxic are biodegraded by an alternative metabolic pathway, thereby decreasing the carcinogenicity of the metabolites, which is desirable for ecological environmental remediation [170].

## 6. Fungi Remediation of Synthetic Dyes: A Biotechnological Alternative for the Treatment of Textile Effluents

The elimination of dyes from textile effluents using biotechnological strategies has emerged as an effective and environmentally sustainable alternative, offering advantages, including low operating costs and a reduced ecological impact [171]. Among the most promising techniques is the use of fungal biomass, which has demonstrated high efficiency in degrading azo dyes. The success of these processes depends mainly on the proper optimization of parameters, such as pH, temperature, humidity, nutrient availability, and culture conditions, which directly affect the enzymatic activity of the fungi involved [172,173].

Recent studies have demonstrated that filamentous fungi utilize a combination of biosorption and enzymatic catalysis to break down azo bonds [174]. For example, *Bjerkandera adusta* strain CX-9 has been reported to produce extracellular peroxidases, such as lignin peroxidase (LiP) and manganese peroxidase (MnP), capable of efficiently degrading dyes such as methyl green, acid blue 158, and Remazol brilliant violet 5R, reaching decolorization rates of 75%, 91%, and 70%, respectively. These results reinforce the potential of fungal enzymes as the key catalysts in bioremediation processes [175].

The degradation of azo dyes by ligninolytic fungi has been the subject of extensive research. A sequential enzymatic mechanism has been described in species such as *Trametes versicolor* and *Phanerochaete chrysosporium* based on two complementary phases. In the first step, fungal azoreductases reduce azo bonds under microaerophilic conditions, generating aromatic amines as intermediate products. Subsequently, in the second phase, ligninolytic laccases and peroxidases oxidize these compounds, promoting their complete mineralization [176,177]. This system has achieved decolorization efficiencies between 90% and 100% for dyes such as reactive black 5 and orange II, with reaction times ranging from 20 to 72 h, depending on whether free or immobilized enzymes or submerged cultures are used [178]. In this context, the use of enzymes immobilized on matrices such as chitosan or alginate has been shown to offer greater operational stability and reusability, being particularly useful for repeated applications in small-scale treatments. In contrast, submerged fungal cultures, although less efficient in enzyme recovery, are more suitable for mass production of enzymes. The optimal conditions for the reported degradation processes include a slightly acidic pH (between 4.5 and 5.0), moderate temperatures (25–30 °C), and dye concentrations ranging from 100 to 500 mg/L [179].

A particularly relevant study demonstrated the catalytic action of manganese peroxidase (MnP), a ligninolytic oxidoreductase secreted by *Phanerochaete chrysosporium* and *Trametes pubescens*, which allows for the oxidation of Mn^2+^ to Mn^3+^ in the presence of hydrogen peroxide (H_2_O_2_) under anaerobic conditions. The generated Mn^3+^ acts as a diffusible redox mediator, capable of breaking azo bonds and degrading aromatic structures of dyes such as reactive black 5 and direct blue 1. This catalytic capacity is supported by the formation of high-valent species, such as the intermediate compounds ferric I (Fe^4+^=O) and II (Fe^4+^–OH), which are essential for the activity of peroxidases. Thus, these enzymes emerge as versatile and highly effective tools for the degradation of persistent synthetic dyes [180]. Table 4 summarizes a representative selection of recent studies using fungi for the degradation of azo dyes, specifying the optimal operating conditions, the decolorization efficiencies achieved, and the enzymatic mechanisms involved in each case.

**Table 4:** Sample of enzymatic degradation studies of azo dyes using filamentous fungi.

Fungi	Enzyme	Azo Dye	Optimal Conditions (pH, T (°C), Absence or Presence of O_2_)	Metabolites/Degraded Products	Mechanism of Action	Efficiency (%)	Ref.
*Fusarium oxysporum*	Laccase Azorreductasa	Reactive Black 5 Orange II	-	Aniline. Phenolic compounds	Oxidation of phenolic groups (laccases) and cleavage of the azo bond (azoreductases)	89.8 91.3	[181]
			30 °C				
			Aerobiosis				
*Aspergillus tamari*	Laccase	Crystal Violet	pH 7	N,N,N′,N′-Tetramethylpararosaniline. 2-(Methylamino)phenol. Benzophenone. 4-methyl amino phenol. 4-(Dimethylamino)benzaldehyde.	Oxidative catalysis of the azo bond	33.8	[182]
		Congo Red	27 °C			74.0	
			Aerobiosis				
*Aspergillus flavus* ASP1	Laccase Lignin peroxidase Quinine reductase	Reactive Orange 16	pH 3	Aniline. 6-(acetylamino)naphthalene-2-sulfonic acid	(Lac/LiP)-mediated azo bond cleavage and detoxification by quinine reductase	100	[183]
			30 °C				
			Aerobic conditions				
*Trametes versicolor*	Laccase	Remazol Red	pH 5	-	Laccase oxidizes the dye by transferring electrons from the substrate (RR) to molecular oxygen, resulting in the formation of water.	54.0	[184]
			45 °C				
			-				
*Peroneutypa scoparia*	Laccase	Acid Red 97	pH 6	-	Reduction of the azo bond	87.5	[185]
			40 °C				
			-				
*Irpex lacteus F17*	Manganese Peroxidase	Malachite green	-	-	Reduction of the azo bond and oxidation of aromatic rings	96.0	
			-				
			-				
*Bjerkandera adusta*		Reactive Blue 120	pH 5	-	Mn^3+^-mediated radical oxidation	90	
			28 °C				
			Aerobic conditions				
*Paraconiothyrium variabile*	Laccase	Acid Red 18	-	-	Oxidative degradation of the azo group	90	
		Direct Red 81	-			68.3	
			-				
RH-2 Consortium *	Laccase Manganese Peroxidase	Congo red	pH 5	-	Enzymatic oxidation of the azo bond	97.1	[186]
			28 °C				
			Aerobic conditions				

* RH-2 Consortium: Penicillium oxalicum (DS-2) and Aspergillus tubingensis (DS-4).

## 7. Photoautotrophic Microalgae and Microbial Consortia in the Biotransformation of Azo Dyes

Photoautotrophic microorganisms, particularly microalgae, have garnered increasing attention as a sustainable and ecologically viable alternative for the remediation of industrial effluents contaminated with synthetic azo dyes [187]. A unique feature of these microorganisms is their ability to harness carbon generated during the mineralization of azo compounds as a primary source of energy, which they integrate into their metabolism [188]. This harnessing capacity minimizes the production of toxic byproducts that remain after the degradation of these pollutants. These autotrophic mechanisms make these technologies eco-friendly tools and also align with the principles of the circular bioeconomy [89].

Furthermore, several microalgae species also exhibit tolerance to high concentrations of azo dyes with strong recalcitrant potential. This resilience, coupled with their enzymatic machinery, gives them a strong potential for cleaving azo bonds. Furthermore, it has been demonstrated that the mechanisms by which degradation occurs generate low-toxicity aromatic amines [37].

Microalgae are two-way agents in wastewater bioremediation, with the advantage of not only degrading artificial dyes, but also exhibiting a pronounced tendency towards adsorption and intracellular accumulation of heavy metals. This is a dual benefit in the case of combined treatment systems, where the removal of organic and inorganic contaminants simultaneously is necessary for effective detoxification [189]. New advances in omics-based approaches and metabolic engineering have already begun to reveal the complex regulatory networks that govern these processes, enabling the rational design of genetically engineered strains with enhanced bioremediation capacity [190].

Microbial consortia have been experimentally shown to also possess the potential to biodegrade a wide variety of azo contaminants. However, their efficiency is strongly dependent on their enzymatic profile. Table 5 reports kinergic consortia consisting of *Chlorella* sp., *Pseudomonas putida*, and *Lactobacillus plantarum*, which have demonstrated efficiency percentages of up to 90% in the degradation of Reactive Blue 40 [191]. This extraordinary biodegradation capacity is largely attributed to the kinergic effects of azoreductases and laccases, enhanced by biosorption processes. These results demonstrate a significant advance in metabolic complementarity against emerging contaminants such as azo dyes [192].

Under more severe conditions, concentrations of up to 500 mg/L of azo contaminants and a pH of 6.5 have been reported. Under these conditions, strains such as *Oedogonium subplagiostomum* (strain AP1) have shown efficiency percentages of up to 97% in the assimilation and degradation of Reactive Orange 122 [193]. Additionally, species such as *Oscillatoria* sp. and *Scenedesmus obliquus* have been reported to have degradation efficiencies of up to 98.5% for Reactive Orange 122 under slightly alkaline conditions (pH 11). These results suggest that these species have developed adaptive abilities that not only enable them to survive in these harsh conditions, but also facilitate their evolution and utilization of dyes as a primary source of carbon [36].

**Table 5:** Examples of the use of different algae strains and consortia in the bioremediation of azo dyes.

Algae Strain/Consortium	Azo Dye	Mechanisms Involved	Metabolites/Degraded Products	Optimal Conditions (Concentration (mg/L), pH, Temperature)	Efficiency (%)	Ref.
*Chlorella vulgaris*	Direct Green 6	Reductive cleavage of azo bonds by azoreductase and decomposition by peroxidases and laccase	-	200 mg/L	78.4	[194]
				pH 8		
				25 °C		
	Direct Black 22	Photodegradation (primary), enzymatic degradation (secondary), and adsorption	-	30 mg/L	-	[194]
				pH 7.2		
				28 °C		
*Oedogonium subplagiostomum AP1*	Methyl orange	Biosorption	Phenols*. Aromatic amines*. Organic acids*.	500 mg/L	97.0	[195]
				pH 6.5		
				30 °C		
*Oscillatoria* sp. y *S. obliquus*	Reactive Orange 122	Enzymatic degradation, adsorption	Cyclic amines*. Phenolic compounds*.	20 mg/L	98.5	[196]
				pH 11		
				25 °C		
*Consortium (Chlorella, Pseudomonas putida,* and *Lactobacillus plantarum)*	Reactive Blue 40	Synergistic degradation: - Azo bond cleavage (azoreductases) - Oxidation of intermediates (laccases/peroxidases) - Adsorption in biomass	Aromatic amines* (typical product of azo bond cleavage). Phenols and modified alcohols*.	1000 mg/L	99.0	[191]
				11		
				35 °C		
*Fucus vesiculosus*	Methyl orange	Biosorption	N^1^,N^1^-dimethylbenzene-1,4-diamine. 4-aminobenzenesulfonate.	57.6	76.8	[197]
				pH 9		
				25 °C		
*Chlamydomonas mexicana*	Red HE8B	Combined enzymatic biodegradation mechanism (Laccases and peroxidases)	N-phenylhydroxylamine. Naphthalen-1-ol. Sodium 5-hydroxynaphthalene-2-sulfonate.	5 mg/L	62	[188]
				pH 7	39	
				27 °C		
	Reactive Green 27					

* The degradation of the dyes was followed by changes in absorption peaks before and after biosorption using FT-IR, indicating the formation of new chemical bonds.

## 8. Yeast-Based Biocatalytic Systems for Azo Dye Degradation: Enzymes, Biosorptive Dynamics, and Biotechnological Potential in Textile Effluent Remediation

Several yeast species have been reported as efficient biotechnological alternatives for the bioremediation of azo contaminants, which is attractive as an eco-friendly alternative to the reported conventional methods [198]. The interest in yeasts stems from their specific mechanisms, which integrate biosorption processes, followed by efficient enzymatic machinery that cleaves azo bonds [199]. The cleavage of azo bonds is so efficient that it does not produce toxic or recalcitrant metabolites. These combined characteristics, coupled with their resilience under extreme environmental conditions, make yeasts promising biotechnological alternatives for ecological environmental remediation methods [185].

Several prominent studies have shown that species such as *Yarrowia lipolytica* and *Candida pseudoglaebosa* exhibit up to 90% efficiency in the biodegradation of persistent pollutants such as RR198; this efficiency is attributed to the high-level endogenous production of oxidoreductases and tyrosinases, which catalyze the degradation of azo dyes by the oxidative cleavage of their chromophore structures. This process converts the complex structures of the dyes into less-complex and/or unstable structures, which facilitates their mineralization by other less-efficient organisms [198,200]. In addition, these species possess an extraordinary adaptive capacity, resisting harsh pH conditions, temperature, and high concentrations of pollutants; these characteristics give them an advantage compared to other microorganisms that are more sensitive [201].

The biocatalytic potential of yeasts is exemplified by the microbial consortium Y-BC-SH, composed of *Yarrowia* sp., *Barnettozyma californica*, and *Sterigmatomyces halophilus* [185]. This consortium achieved complete decolorization of Reactive Black 5 within three hours, a result driven by the synergistic activity of laccases, manganese peroxidases (MnP), and azoreductases (AzoR) (Table 6). This metabolic–enzymatic synergism, combined with the stability and adaptive resilience of the various strains reported, indicates the relevance and viability of these highly efficient biotechnological alternatives [201].

A representative case is *Geotrichum candidum*, which generates DyP class peroxidases that degrade methyl orange up to 94.6% at high concentrations [202]. The generated metabolites are not completely degraded, and this may require a synergistic contribution from another species that degrades these metabolites. Similarly, *Galactomyces geotrichum* KL20A demonstrated 76.6% removal of methylene blue at 35 °C and 50 mg/L, whereas the HYC consortium (*Sterigmatomyces halophilus* and *Meyerozyma guilliermondii*) achieved 96.1% removal within 48 h, mediated by NADH-DCIP reductases [203]. These findings reinforce the importance of selecting metabolically compatible and enzymatically potent strains for biotechnological applications [201].

Among the most efficient yeast species reported are *Cyberlindnera fabianii*, *Candida tropicalis* A1, *Candida zeylanoides*, and *Galactomyces geotrichum* (strain MTCC 1360), all of which achieved degradation rates exceeding 97% in less than 12 h. Notably, *Galactomyces geotrichum* demonstrated complete degradation of methyl red within one hour at pH 3, while *Candida tropicalis* A1 achieved 97.5% degradation of Acid Red B, producing fewer recalcitrant metabolites such as 4-amino-naphthalene-1-sulfonic acid. The Y-BC-SH consortium also demonstrated complete degradation of Reactive Black 5 within three hours, facilitated by the combined activity of lipases, xylanases, and ligninolytic enzymes [204,205,206].

**Table 6:** Reports of synthetic dye degradation by yeasts and microbial consortia.

Yeast Strain/Consortium	Dye	Mechanisms Involved	Metabolites/Degraded Products	Conditions (Time (h), pH, T (°C), Dye Concentration (mg/L))	Removal/ Decolorization (%)	Ref.
*Cyberlindnera fabianii*	Acid Red 14	Laccase (Lac), Tyrosinase (Tyr), Manganese Peroxidase (Mnp), Azoreductase (AzoR)	-	12 h	97	[205]
				pH 5		
				30 °C		
				50 mg/L		
*Saccharomyces cerevisiae*	Acid Orange 7	Biosorption (Immobilization in Fe_3_O_4_)	-	2.3 h		
				pH 6.5		
				35 °C		
				50 mg/L		
	Violet crystal	The enzymes involved are not identified.	-	24 h	84.9	[203,207]
				pH 7		
				30 °C		
				1000 mg/L		
*Pichia kudriavzevii* SDG12	Reactive Black 5	Azoreductase	Unspecified amines and aromatic compounds	18 h	100	[208]
				pH 7		
				32 °C		
				100 mg/L		
*Candida tropicalis* A1	Acid Red B	Azoreductase (AZR), Laccase (Lac), Manganese peroxidase (MnP), Lignin peroxidases (LiP)	4-amino-naphthalene-1-sulfonic acid, 4-hydrazinylnaphthalene-1-sulfonic acid, naphthalene-1,2,4-triol, 1-phenylethenol	12 h	966	[209]
				pH 7		
				30 °C		
				70 mg/L		
*Galactomyces geotrichum* MTCC 1360	Methyl Red	-	2-Aminobenzoic acid N,N-Dimethyl-*p*-phenylenediamine	1 h	100	[204]
				pH 3		
				30 °C		
				100 mg/L		
*Sterigmatomyces halophilus* SSA-1575	Reactive Black 55	NADH-DCIP reductase Lignin peroxidase (LiP) Azoreductase	Catechol cis-9-octadecenoic acid Aniline 4-methylsulfonyl aniline Benzene 2-(4′-aminophenyl) sulfonyltholNaphthalene-1,2,4-triol2,7,8-triaminonaphthalenol	12 h	98.7	[210]
				pH 5		
				30 °C		
				750 mg/L		
*Candida zeylanoides*	Reactive Orange 16 (RO16)	Reductases (azoreductase, NADH-dichlorophenolindophenol reductase)	4-(Methyl sulfonyl)aniline, α-Hydroxybenzene propanoic acid	5 days	100	[206]
				-		
				28 °C		
				150 mg/L		
Mixed consortium (*Pleurotus ostreatus* and *Candida zeylanoides*)	Reactive Orange 16 (RO16)	Manganese peroxidase (MnP), Laccase	4-(Ethenyl Sulfonyl) benzene, (Methylsulfonyl) benzene, 2-(Phenyl Sulfonyl) ethanol, 4-(Ethenyl Sulfonyl) aniline, α-Hydroxybenzenepropanoic acid	11 days	87.5	
				-		
				28 °C		
				150 mg/L		
Y-BC-SH Consortium *	Reactive Black 5	Lipase, Xylanase, Laccase, Azoreductase, LiP, MnP	2,7,8-triaminenonaphthalen-1-ol, 2-chloro-4,6-diamino-1,3,5-triazine, aniline, 2-naphthol, lauric anhydride	3 h	100	[185]
				pH 8		
				18 °C		
				200 mg/L		
HYC Consortium **		NADH-DCIP reductase, azoreductase, veratryl alcohol oxidase, aldehyde dehydrogenase	1,3,5-Trimethylbenzene (TMB), benzoic acid (BA), 2,4-Di-tert-butyl phenol (DTBP)	48 h	96.1	[201]
				pH 7		
				35 °C		
				50 mg/L		

* Y-BC-SH: *Yarrowia* sp. SSA1642, Barnettozyma californica SSA1518, *Sterigmatomyces halophilus* SSA1511. ** HYC: Dominated by *Sterigmatomyces halophilus* SSA-1575 and *Meyerozyma guilliermondii* SSA-1547.

## 9. Conclusions

Synthetic dyes, particularly azo dyes, are abundant and widely used, with applications in a diverse range of industries worldwide. Of these dyes, more than 7000 variants are estimated to be commonly used in influential industrial sectors. It has been identified that the discharge of industrial effluents with high azo compound loads is generating environmental damage that is difficult to reverse. This is problematic because these compounds alter key parameters, such as pH, oxygen levels, and dissolved carbon levels, which are essential to maintaining ecological balance. Furthermore, the formation of recalcitrant and toxic intermediates poses an imminent threat to human and animal health. Given this situation, several remediation strategies have recently been reported, among which high-efficiency biotechnological processes stand out. More information is needed on the synergistic effects between species that enhance the mineralization of these pollutants, while minimizing the production of toxic intermediates.

Given the effects of the growing environmental pressure generated by these pollutants, biotechnological applications are attracting significant attention as efficient and eco-friendly strategies with the potential to decontaminate industrial effluents with high azo contaminant loads. These organic compounds, whose robust molecular structures confer extraordinary environmental resilience, challenge the traditional and standard physicochemical methods for treating these effluents, finding biotechnological methods a highly robust and efficient alternative. An interesting example is yeast, which not only demonstrates remarkable tolerance to harsh conditions, but also deploys a diverse enzymatic repertoire (laccases, peroxidases, and azoreductases) combined with biosorption processes capable of degrading complex chromophore structures with high efficiency. Furthermore, microorganisms such as *Pseudomonas putida* and *Bacillus cereus*, fungi such as *Trametes versicolor* and *Aspergillus flavus* and microalgae such as *Chlorella vulgaris* and *Lychaete pellucida* have demonstrated complementary capacities to remove these contaminants, each adapted to specific niches of pH, temperature, and concentration, which could exhibit synergistic effects with microorganisms that present limitations in a particular condition. This functional diversity not only expands the range of applications, but also invites us to rethink bioremediation as a cooperative process, where synergy between species can overcome individual limitations. In this sense, microorganisms are positioned not only as mere technical agents, but also as silent, highly efficient allies in restoring an ecological balance that has been deeply affected by the uncontrollable fashion and color industries.

## Figures and Tables

**Table 1:** Steps of PICO methodology for designing search keywords.

STEP 1	Study Idea	Degradation of Azo Dyes by Aquatic and Terrestrial Microorganisms					
STEP 2	Study problem	P	Azo dyes				
		I	Degradation/removal of azo dyes by microorganisms such as yeast, bacteria, fungi, microalgae, and consortia				
		C	Degradation/removal of azo dyes by conventional treatments				
		O	Bioremediation				
STEP 3	Research question	Can biodegradation based on yeast, bacteria, fungi, microalgae, and consortia offer a viable and environmentally friendly solution for detoxifying aquatic ecosystems severely affected by persistent azo dyes?					
STEP 4	DeCS	Azo compounds		DeCS			
		Accumulation in water and land		Soil*, Water*			
		Aquatic and terrestrial microorganisms		Microorganism*			
		Bioremediation		Bioremediation (Environmental Health)			
STEP 5	MeSH similarity in PUBMED	Azo dyes	Azo Compounds*	Compounds, Azo*	Dyes, Azo*	Azo Dye*	Dye, Azo
		Soil*, Water*	Soil*	Water*	Aquatic*		
		Microorganism*	Microbe*	Microbiota*	Bacteria*	Fungi*	Microbiome*
		Bioremediation	Degradation*	Biodegradation*	Depolymarization*	Mineralization*	Assimilation*
STEP 6	Search approach by variables	Azo dye	Azo dyes* OR Azo Compounds* OR Compounds, Azo* OR Dyes, Azo* OR Azo Dye* OR Dye, Azo				
		Biodegradation, Environmental	Soil* OR territorial* OR land* OR water* OR aquatic*				
		Microorganism	Microbe* OR microorganism* OR microbiota* OR microbiome* OR bacteria* OR fungi* OR biofilm*				
		Bioremediation	Degradation* OR biodegradation* OR depolymerization* OR mineralization* OR assimilation*				
STEP 7	Advanced search key	(Azo dyes* OR Azo Compounds* OR Compounds, Azo* OR Dyes, Azo* OR Azo Dye* OR Dye, Azo) AND (soil* OR territorial* OR land* OR water* OR aquatic*) AND (microbe* OR microorganism* OR microbiota* OR microbiome* OR bacteria* OR fungi* OR biofilm*) AND (degradation* OR biodegradation* OR depolymerization* OR mineralization* OR assimilation*)					

* It is used to indicate in the search that the root of the word should be recognized regardless of whether it is plural or singular or the gender of the term.

**Table 2:** General characteristics of the most used physicochemical methods for dye removal.

Method/Technique	Removal Mechanism	Advantages	Disadvantages *	Ref.
Adsorption	Dye removal by adhesion to the surface of an adsorbent	Reuse of adsorbents, high efficiency, and short times for removing dye from wastewater	-Only soluble dyes. -High energy consumption.	[120,121,122]
Ion exchange	Use of resins that allow ionic exchange between the substances involved	The ion exchange process can effectively remove cationic dyes from contaminated water	The resulting sludge may contain concentrated metals, posing challenges for disposal	[123,124]
Coagulation and flocculation	In this process, coagulants are used to destabilize dissolved dyes, enabling their removal through sedimentation.	Coagulation–flocculation is a simple and widely used process for removing dyes from wastewater.	The initial pH and the dosage of the coagulant have a significant influence on the coagulation and flocculation process.	[125,126]
Membrane filtration	They use membranes with small pores that trap solutes larger than themselves, allowing the passage of a dye-free solution	-High separation efficiency, reliability, cost-effectiveness, and simplicity. -They have low operating costs compared to conventional technologies.	-Production of toxic byproducts. -Sludge production.	[127,128]

* Pore fouling is another critical disadvantage of membrane filtration systems. This technical limitation affects the operational efficiency and lifespan of the systems.

## Data Availability

The data presented in this study are openly available in this article.
